# In vitro characterization of AA71, a potent and selective human monoclonal antibody against CGRP receptor

**DOI:** 10.1186/1129-2377-14-S1-P183

**Published:** 2013-02-21

**Authors:** L Shi, S Rao, H Sun, K Wild, C Xu

**Affiliations:** 1 Department of Neuroscience, Amgen, Inc. Thousand Oaks, California, USA

## Objectives

To characterize the in vitro pharmacological properties of AA71, a human monoclonal antibody against the CGRP receptor.

## Introduction

Calcitonin gene-related peptide (CGRP) is a neuropeptide that plays a key role in the pathophysiology of migraine. Clinical studies have demonstrated that CGRP receptor antagonism is an effective approach in treating acute migraine pain. We have previously reported the successful generation of a group of human monoclonal antibodies (MAb) that specifically target the human CGRP receptor ( 1 ). In our present study, we detail the characterization of AA71, a potent and selective human monoclonal antibody against the CGRP receptor.

## Results

AA71 potently competed with the binding of [125I]-CGRP to the human CGRP receptor with a Ki of 0.03 nM. AA71 fully inhibited CGRP-stimulated cAMP production with an IC50 of 2 nM in cell-based functional assays, with no intrinsic agonist activity up to 10 ƒÝM. Functional potency of AA71 at the cyno CGRP receptor was similar to that at the human receptor with an IC50 of 4.5 nM, but potency at dog, rabbit and rat receptors was significantly reduced (>5000-fold). AA71 also demonstrated > 5000-fold selectivity over other closely related receptors in the family. Through a saturation analysis using SK-N-MC membrane preparations, [125I]-AA71 was shown to bind the CGRP receptor in a monophasic and saturable fashion with a Kd of 0.08 nM. The binding of 0.1 nM [125I]-AA71 to the CGRP receptor reached equilibrium at approximately 240 min. A prolonged dissociation of [125I]-AA71 binding from the CGRP receptor was observed with a dissociation t1/2 off of 267 min. In a competition study, CGRP¡¦s capability of displacing [125I]-AA71 binding is significantly less robust, with an observed Ki of 370 nM.

## Conclusion

AA71 is a potent and selective antibody against the human CGRP receptor with potential for use in the treatment of migraine pain.
